# Exploring the Relationship Between Susceptibility to Health Misinformation and Vaccine Hesitancy in Poland

**DOI:** 10.3390/healthcare14040497

**Published:** 2026-02-14

**Authors:** Mariusz Duplaga, Magdalena Sikorska, Urszula Zwierczyk, Kinga Kowalska-Duplaga

**Affiliations:** 1 Department of Health Promotion and E-Health, Institute of Public Health, Faculty of Health Sciences, Jagiellonian University Medical College, Skawińska Str. 8, 31-066 Krakow, Poland; magdalena1.sikorska@uj.edu.pl (M.S.); urszula.zwierczyk@uj.edu.pl (U.Z.); 2 Department of Pediatrics, Gastroenterology and Nutrition, Medical Faculty, Jagiellonian University Medical College, Wielicka Str. 265, 30-663 Krakow, Poland; kinga.kowalska-duplaga@uj.edu.pl

**Keywords:** vaccine hesitancy, vaccine conspiracy beliefs, health misinformation, susceptibility to misinformation, health literacy, e-health literacy, trust in scientists

## Abstract

**Background/Objectives:** Vaccine hesitancy arises from multiple determinants, including individual beliefs, cognitive style, social norms, political identity and the information environment. In this context, health literacy, e-health literacy, susceptibility to health misinformation, conspiracy beliefs and trust in science may be relevant in mediatized societies. **Aim:** The aim of the study was to examine how susceptibility to health misinformation relates to vaccine hesitancy in Poland and how this association is influenced by health literacy, e-health literacy, trust in scientists and sociodemographic factors. **Methods:** Data came from a web-based survey conducted in December 2024 among 2200 adults aged 18–75 years. The questionnaire included validated scales of vaccine hesitancy, health literacy, e-health literacy, vaccine conspiracy beliefs and trust in scientists. The susceptibility to health misinformation was measured with ad hoc instrument based on the statement from fact-checking services. Items assessing digital media use, political sympathies, religious practices and sociodemographics were also applied. Multivariable linear regression was applied with continuous vaccine hesitancy as the dependent variable. **Results:** The model explained 57.8% of the variance in vaccine hesitancy. Susceptibility to misinformation (B = 0.11, 95% CI: 0.08–0.15) and vaccine conspiracy beliefs (B = 0.44, 95% CI: 0.41–0.46) were positive predictors, whereas trust in scientists (B = −0.20, 95% CI: −0.23–−0.17) and e-health literacy (B = −0.07, 95% CI: −0.11–−0.02) were protective. Older age was associated with lower hesitancy (B = −0.02, 95% CI: −0.03–0.00). Secondary education (B = −0.58) and a master’s degree (B = −0.77) predicted lower hesitancy. Health literacy categories were not significantly related to vaccine hesitancy. **Conclusions:** Susceptibility to health misinformation and vaccine conspiracy beliefs were key predictors of vaccine hesitancy, outweighing the effects of health literacy and the protective impact of trust in scientists and e-health literacy, and indicating a need for interventions that combine prebunking and literacy-focused strategies with efforts to strengthen confidence in health institutions.

## 1. Introduction

Vaccine hesitancy (VH) is used to describe attitudes that range from full acceptance of vaccines to outright refusal. It reflects that vaccination decisions depend not only on access to services, but also on psychological factors, social influences, and the information people receive. Conceptual frameworks emphasize confidence, perceived risk, practical barriers, social norms, deliberate weighing of benefits and harms, and openness to conspiracy-type explanations, showing that hesitancy is linked to wider cultural and political conditions. In digital settings, misleading vaccine claims and denialist messages may weaken trust in science and public institutions and may change how people judge risks, which can increase hesitancy. Susceptibility to such content is related to health (HL) and e-health literacy (eHL), cognitive style, political orientation, and trust in scientists. Against this background, we asked whether susceptibility to health misinformation (SHM) is associated with VH in Poland after accounting for vaccine conspiracy beliefs (VCB), trust in scientists, HL, eHL, political sympathies, religious practices and sociodemographic factors.

Over the last twenty years, numerous conceptual tools have been proposed to describe how individuals and communities relate to vaccination, including constructs such as vaccine confidence, perceived risk, and broader categories of acceptance and refusal [1,2]. Among these, the notion of VH has become particularly prominent in empirical research and public health debates because it captures the nuanced and fluid range of attitudes situated between unquestioning acceptance and categorical rejection of vaccines [3]. The Strategic Advisory Group of Experts (SAGE) of the World Health Organization characterizes VH as a postponement or rejection of vaccination despite the ready availability of vaccination services [3], thereby emphasizing that hesitancy is distinct from outright opposition and reflects a spectrum of doubts, ambivalence, and situational factors.

As an analytical construct, VH has gained traction precisely because it reflects the complex determinants of vaccination behavior, which extend beyond individual knowledge or beliefs to include social norms, cultural and political contexts, and the pervasive influence of misinformation [4,5]. It offers a framework for understanding how concerns about vaccine safety, perceived necessity, and distrust toward institutions intersect to shape uptake. In doing so, the concept highlights that obstacles to vaccination are psychological and informational as well as structural. Focusing on VH enables researchers and policymakers to identify groups at heightened risk of delaying or foregoing vaccination and to design targeted interventions that address their specific doubts and constraints, thereby helping to close the gap between vaccine availability and actual coverage [6].

The 3C, 5C, and 7C frameworks map key psychological drivers of VH along a continuum from acceptance to refusal. They are conceptual models developed to organize the main determinants of vaccination decisions and to support measurement (e.g., survey constructs), risk group profiling, and the design of targeted interventions. In practice, they also help distinguish whether hesitancy is driven mainly by trust-related concerns, perceived risk, access barriers, social norms, or beliefs shaped by the information environment. The 3C model identifies confidence (trust in vaccines, providers, policy), complacency (low perceived disease risk), and convenience (availability and affordability) as primary determinants [3]. The 5C model refines this by replacing convenience with constraints (practical barriers) and adding calculation (deliberative risk–benefit weighing) and collective responsibility (protecting others via herd immunity), which helps separate access-related barriers from cognitive processes and prosocial motives [5]. The 7C model is a validated extension used to assess vaccination readiness and predict hesitancy: it retains confidence, complacency, constraints, calculation, and collective responsibility, and adds compliance (norms/obedience to authorities) and conspiracy (propensity to endorse misinformation) [7]. Importantly, the domains are not mutually exclusive. The same individual may experience practical barriers and, at the same time, hold doubts linked to trust or conspiratorial interpretations. The frameworks are also not mechanistic models of causality. They provide a consistent vocabulary for describing and measuring determinants and for deciding which levers are most relevant for communication and service-delivery strategies. Together, these models show that hesitancy arises not only from trust and access, but also from deliberation, social norms, and information ecosystems. In the present study, this framework informed our focus on variables aligned with the “conspiracy” and “confidence” domains (susceptibility to misinformation, vaccine conspiracy beliefs, and trust in scientists), while also accounting for literacy and key sociodemographic correlates.

VH arises from a complex interplay of individual, social, and contextual factors. Sociodemographic predictors include age, gender, education, and socioeconomic status, with lower education and income often associated with greater hesitancy [8]. Psychological antecedents include distrust in authorities, fear of side effects, low perceived disease risk, and reliance on anecdotal evidence [9]. Health literacy and e-health literacy play crucial roles. Individuals with higher health literacy, defined as the ability to access, understand, appraise, and apply health information, are less likely to be vaccine hesitant [8]. E-health literacy, the ability to evaluate online health information, becomes particularly important in digital environments rife with misinformation [10]. E-health literacy mediates the relationship between educational level and vaccine attitudes, with individuals possessing higher e-health literacy demonstrating more favorable vaccine beliefs regarding susceptibility, severity, and benefits [11].

Cognitive style also matters. People with stronger reflective or analytical thinking, as measured by cognitive reflection tests, are less likely to accept misinformation at face value and tend to have lower VH [12,13,14]. Conversely, intuitive cognitive styles are linked to susceptibility to conspiracy beliefs and higher hesitancy [12].

Political ideology influences vaccine attitudes as well. In several countries, conservative or populist sympathies correlate with greater mistrust in science and vaccines [15,16,17,18]. These political identities intersect with exposure to misinformation, as partisan media ecosystems selectively amplify certain narratives.

Misinformation has become one of the defining challenges of contemporary public health. The term misinformation generally refers to false, inaccurate, or misleading information that is spread without necessarily intending to deceive [19,20]. In contrast, disinformation describes deliberately false information intended to mislead or manipulate [19], while malinformation refers to information that is technically accurate but is presented or used in misleading, harmful ways [21]. So, from a functional perspective, misinformation can be unintentional, spread by individuals who genuinely believe it or intentional disinformation deployed strategically for political, ideological, or economic gain [22]. The contemporary information environment, characterized by global connectivity, social media platforms, and rapid information exchange, has dramatically increased the reach, speed, and impact of misinformation [23]. Therefore, digital platforms became the most important dissemination channel of misinformation, apart from traditional media and intrapersonal networks. Social media plays a pivotal role, enabling rapid viral spread of health misinformation and creating echo chambers that reinforce prior beliefs [23,24]. Lewandowsky et al. (2017) emphasized the role of “post-truth” communication, where emotional narratives and identity-driven beliefs often outweigh factual accuracy [25].

Health misinformation refers to health-related claims that are currently false due to a lack of scientific evidence [26]. Such claims can be categorized into inaccurate claims about the causes of diseases, the effectiveness or safety of medical treatments, preventive measures, and vaccines [20,27]. Common themes of health misinformation include vaccine safety, side effects, the necessity of preventive measures, the promotion of alternative remedies, and distrust in authorities [28,29]. Vaccine misinformation can take two primary forms: context misinformation (misleading statistics, overgeneralized anecdotal evidence) and content misinformation (false or fabricated claims and conspiratorial narratives), with context misinformation being more prevalent [30]. Importantly, the line between information and misinformation is often blurred when scientific knowledge is uncertain or evolving [31].

In the domain of health, misinformation has particularly dangerous implications, affecting risk perceptions, trust in institutions, and ultimately health behaviors with reduced adherence to preventive measures, and even leading to harmful self-treatment practices [20,32,33,34]. The COVID-19 pandemic illustrated the profound impact of health misinformation on vaccine acceptance, preventive behavior adoption, and trust in medical science [34,35]. During the COVID-19 pandemic, it was emphasized that widespread falsehoods undermined pandemic response efforts, distorted risk perceptions, and fostered distrust [33]. The global infodemic during COVID-19 also showed how misinformation spreads faster than corrective information and can dominate public discourse [23].

Health misinformation is closely linked to denialism, which is the systematic rejection of well-established scientific consensus [36]. Denialism differs from ordinary misinformation by being ideological, motivated, and resistant to correction. For instance, climate change denial or vaccine denial are grounded not only in false claims but also in organized communities that reject mainstream evidence, often invoking conspiracy narratives [25]. Vaccine denialism exemplifies this process: it selectively amplifies misinformation about vaccine risks, disregards corrective evidence, and frames consensus science as corrupt or politically motivated [9,37]. Such denialist orientations are often more resistant to debunking because they are tied to identity, distrust in institutions, and political ideology [38,39].

Vaccines have been among the most frequent targets of health misinformation. Vaccine misinformation ranges from inaccurate claims about side effects (e.g., vaccines cause autism), exaggerated risks (e.g., infertility, DNA alteration), to conspiratorial claims that vaccines are profit-driven plots by pharmaceutical companies [35,40]. Broniatowski et al. (2018) demonstrated that malicious actors, including state-sponsored trolls and bots, intentionally amplified divisive vaccine discourse online, illustrating the geopolitical stakes of misinformation [22].

Denialism thrives in the same information environments as misinformation. Exposure to falsehoods repeatedly can normalize them, a phenomenon known as the “illusory truth effect” [41]. As misinformation accumulates, it fosters generalized distrust in scientific expertise, providing fertile ground for denialist belief systems. Consequently, understanding susceptibility to misinformation is central to explaining how denialism emerges and how it undermines public health strategies.

The effects of vaccine misinformation are profound. Research showed that exposure to anti-vaccine conspiracy theories reduces vaccination intentions [40] and vaccination uptake [42]. Misinformation exposure during COVID-19 significantly decreased willingness to vaccinate and increased VH and refusals [35,43,44,45]. Social media are usually perceived as the main channel of spreading misinformation, also in relation to vaccination [46]. The analysis of the data from a multinational survey revealed that there is a significant relation between heavy use of social media or its use as a primary source of news and VH [47].

VH is not identical to vaccine refusal or denialism, but they are related. Hesitancy often represents a middle ground between acceptance and refusal, whereas denialism involves outright rejection of vaccines and underlying science [48]. However, misinformation contributes to both phenomena. Repeated exposure to misinformation erodes trust, fosters conspiratorial thinking, and can shift hesitant individuals toward denialist positions [25,40].

Health denialism substantiated by conspiracy beliefs amplifies misinformation by reframing scientific consensus as ideology, suggesting that vaccines are promoted for profit or control rather than health. This rhetorical strategy transforms misinformation into identity-affirming narratives that resist correction [9]. Consequently, both susceptibility to misinformation and denialist orientations are critical to explaining VH.

Susceptibility to misinformation refers to the degree to which individuals believe, accept, or fail to critically evaluate false claims [49]. It is influenced by cognitive style, trust in science, literacy, political ideology, and emotional predispositions. Globally, susceptibility to COVID-19 misinformation predicted reduced vaccination intent and lower compliance with public health guidance [49]. Experimental studies suggest that even brief exposure to misinformation can significantly alter attitudes toward vaccination [35].

Cognitive reflection and rational thinking education can mitigate susceptibility [50]. However, high education does not immunize individuals; motivated reasoning and identity-protective cognition can sustain susceptibility even among educated populations [25]. Thus, susceptibility is not only a function of knowledge but also of trust, identity, and motivation.

Given the centrality of misinformation in shaping vaccine attitudes, the present study aims to assess the relationship between VH and SHM among adults. In addition, the study examines how HL, eHL, political sympathies, and trust in scientists interact with SHM and VCB in predicting VH. This multidimensional approach allows us to capture the complex determinants of hesitancy, integrating cognitive, informational, and political domains.

Building on this framework, we hypothesize that greater SHM and more intense VCB will be positively associated with higher VH, while higher HL, eHL, and trust in scientists will be negatively related to hesitancy. Furthermore, we expect political sympathies will be significantly associated to VH, with stronger effects observed among individuals whose ideological orientations involve distrust of scientific institutions.

## 2. Materials and Methods

### 2.1. Survey

In this study, we analyzed the data collected through a computer-assisted web-based interviewing (CAWI) survey conducted on a representative sample of 2200 adult Internet users aged 18–75. The sample was adjusted for age, education, gender, place of residence, and NUTS1 region to reflect the structure of the adult Internet-using population in Poland in 2023, as reported by Statistics Poland, the national statistical bureau [51]. The target sample size was predefined based on feasibility and available funding, while aiming to ensure stable estimates across quota strata and sufficiently sized subgroups for planned analyses. At a confidence level of 0.95 and a fraction of 0.5, the approximate margin of error was 2.1% under simple random sampling assumptions (assuming that the population of Internet users aged 18–75 surpassed 26,000,000 in 2023 [51]). The survey was carried out in December 2024 by Fieldstat LLC, a company specializing in online studies of public opinion research [52]. Respondents invited to participate were recruited from the National Research Panel, maintained by the company and comprising 37,000 active panelists (23). Eligible participants were panel members who were adult internet users aged 18–75 years residing in Poland and who provided informed consent. Respondents were excluded if they did not meet eligibility criteria, declined participation, or submitted incomplete questionnaires.

The study was approved by the Bioethical Committee of Jagiellonian University (Decision No 118.0043.1.271.2024 from 26 September 2024, with amendments). Individuals invited to participate in the survey were informed about the study’s objectives and anticipated outcomes. Informed consents were obtained electronically prior to granting access to the online questionnaire.

### 2.2. Questionnaire

The questionnaire applied in the survey consisted of 114 individual items, including the following instruments: the 10-item Adult Vaccine Hesitancy Scale [53,54] the 12-item Health Misinformation Susceptibility Questionnaire, the 6-item European Health Literacy Survey Questionnaire (HLS-EU-Q6) [55], the 10-item e-Health Literacy Scale (eHEALS) ([56,57]), the 7-item Vaccine Conspiracy Beliefs Scale [42,58] the 12-item Trust in Scientists Scale [59]. Apart from these tools, the questionnaire encompassed a set of items asking about the use of Internet and social media, political sympathies, religious practices, demographic and socioeconomic characteristics.

### 2.3. Instruments

#### 2.3.1. Adult Vaccine Hesitancy Scale

The 10-item Adult Vaccine Hesitancy Scale (aVHS) is an adaptation of the original WHO SAGE Vaccine Hesitancy Scale for parents [60], reworded by Akel et al. (2021) so that all items refer to respondents’ own adult vaccinations rather than their children’s [53]. In Poland, Duplaga et al. translated and culturally adapted the aVHS following standard forward–backward translation, expert-panel review and cognitive interviews, and then validated the Polish version (PL-aVHS) in a large online sample of adults [55]. The scale consists of 10 statements covering confidence in vaccines, perceived risks, and attitudes toward new vaccines. Each item is answered on a 5-point Likert scale from “strongly disagree” (1) to “strongly agree” (5), with several items reverse-coded so that higher values uniformly reflect greater hesitancy. Item scores are summed to give a total between 10 and 50. In our study, internal consistency of the scale was confirmed with high Cronbach’s alpha coefficient equal to 0.91.

#### 2.3.2. Susceptibility to Health Misinformation Instrument

The Health Misinformation Susceptibility Instrument (HMSI) is an ad hoc measure developed to assess individuals’ ability to correctly evaluate the veracity of health-related statements circulating in the Polish information environment in post-pandemic era. The decision to construct an ad hoc instrument was driven by the need to ensure that the items closely reflect currently circulating health-related misinformation in Poland, thereby increasing ecological validity and the contextual relevance of the measure. Using contemporary and widely disseminated false claims also maximizes the likelihood that respondents have already encountered similar content in their everyday media use, enabling the assessment to approximate real-world evaluative processes rather than reactions to unfamiliar or outdated statements.

The construction of the instrument was inspired by approaches used in prior research on susceptibility to misinformation and conspiratorial claims, where respondents judge the truthfulness of empirically verifiable statements rather than report abstract attitudes [34,61,62]. In the first step, a pool of over 50 fake news items was established based on the content of the main fact-checking services available in Poland. The authors’ reviewed this pool for relevance, recognizability, and clarity, and excluded statements that were clearly implausible or trivially false, in order to avoid ceiling effects and to ensure sufficient variance in responses. To assure appropriate recognizability only the fake news identified by fact-checking services in 2023–2024 were included.

In the next step, the number of fake news items was reduced to 20 to capture prevalent false beliefs that had gained substantial traction in the Polish infosphere [63]. In a final step, only 12 items were selected to assure timeliness, appropriate thematic representativeness, and broad recognizability in the general population, while excluding niche statements that would be specific to narrow subgroups (e.g., specialized misinformation about statin therapy relevant to patients suffering from cardiovascular diseases). On the basis of these 12 fake news items, false statements covering diverse health-related domains (e.g., vaccines, lifestyle, treatment practices) were developed. The tool was piloted in a group of 10 respondents drawn from the general population. The pilot was used to verify the clarity and feasibility of the instrument and suggested that the items were understandable and elicited sufficient variability in responses, thus supporting its preliminary usefulness for further survey research [62]. The structure and origin of individual items included in the HMSI is available in Table S1.

Respondents are asked to indicate the perceived truthfulness of each statement on a 6-point Likert scale ranging from “decidedly false” (1) to “decidedly true” (6), which allows for a nuanced assessment of gradations in belief rather than a simple dichotomous true/false response. Item responses are coded so that higher scores reflect greater susceptibility to misinformation, and a total score is calculated as the sum of all item scores, yielding a possible range from 12 to 72. The Instrument showed high internal consistency (Cronbach’s α = 0.81). Factorial validation of the HMSI (polychoric EFA, forced one-factor EFA, and benchmark CFA models with relevant results in Table S2.1–S2.10) is reported in Supplementary File S2.

#### 2.3.3. European Health Literacy Survey Questionnaire

The 6-item European Health Literacy Survey Questionnaire (HLS-EU-Q6) is a short form derived from the 47-item HLS-EU-Q developed by Sørensen et al. within the European Health Literacy Survey Project [55]. It selects six items that summarize the core construct of general health literacy: the perceived difficulty of finding, understanding, judging and using health information in healthcare, disease prevention and health promotion contexts. Each item is answered on a 4-point Likert scale with response options “very difficult,” “fairly difficult,” “fairly easy,” and “very easy,” which are typically coded from 1 to 4. A health literacy index is calculated as the mean of the available item scores requiring at least five of six items to be answered, so that higher values represent better health literacy. Conventional cut-offs classify mean scores ≤2 as “inadequate,” >2 to 3 as “problematic,” and >3 as “sufficient” health literacy [64]. The scale demonstrated adequate internal consistency (Cronbach’s alpha = 0.82).

#### 2.3.4. e-Health Literacy Scale

The e-Health Literacy Scale (eHEALS) was originally introduced by Norman and Skinner (2006) as an 8-item self-report measure of perceived skills in finding, evaluating and applying health information from the Internet [57]. In many later applications, two general items about the perceived usefulness and importance of online health information are included alongside the eight core competence items, so that respondents answer 10 statements in total. The Polish version (Pl-eHEALS), adapted and validated by Duplaga et al., was developed using WHO-style transcultural procedures: independent forward translations by domain experts, reconciliation by an expert panel, back-translation, and cognitive interviews with both younger and older adults [56]. All items are rated on a 5-point Likert scale from “strongly disagree” (1) to “strongly agree” (5). Scoring following the original authors’ recommendation sums the eight core competence items, giving a total score from 8 to 40, where higher scores indicate higher perceived e-health literacy; the two introductory items, when present, are often analyzed descriptively but not included in the total. Cronbach’s alpha coefficient was equal to 0.88.

#### 2.3.5. Vaccine Conspiracy Beliefs Scale

The 7-item Vaccine Conspiracy Beliefs Scale (VCBS) was developed by Shapiro et al. (2016) by adapting six explicitly conspiratorial items from Jolley and Douglas’ work on vaccine conspiracy theories [40] and adding one new item, yielding a brief unidimensional measure [58]. The items capture beliefs that powerful actors hide vaccine risks, fabricate safety data, or use vaccines for harmful ulterior motives. All seven items are rated on a 7-point Likert scale from “strongly disagree” (1) to “strongly agree” (7). Scores are summed to obtain a total between 7 and 49, with higher scores reflecting stronger endorsement of vaccine conspiracy beliefs; because all items are phrased in the conspiratorial direction, no reverse-coding is needed. Kowalska-Duplaga and Duplaga translated and adapted the VCBS (PL-VCBS) to Polish using forward–backward translation, expert review and piloting with cognitive interviewing, and then validated it in a large national online panel [42]. Their validation confirmed a one-factor structure and excellent internal consistency of the scale. The Scale had a high internal consistency (Cronbach’s alpha = 0.94).

#### 2.3.6. Trust in Scientists Scale

The 12-item Trust in Scientists Scale attributed to Cologna et al. (2025) is a very recent instrument designed to capture general trust in scientists as a social group, building on earlier “trust in science and scientists” inventories [59]. It typically includes items tapping perceived honesty, competence, integrity, benevolence and motivations of scientists, e.g., whether scientists tell the truth about risks, put societal welfare above personal or corporate interests, and follow rigorous methods. The items are answered on a 5-point agreement Likert scales relevant to each item. Scoring is based on computing the sum across all 12 items. This Scale also showed high internal consistency in our study (Cronbach’s alpha = 0.91).

### 2.4. Statistical Analysis

All statistical analyses were conducted using the latest version of IBM SPSS Statistics v.29 (IBM Corp., Armonk, NY, USA). Descriptive statistics were used to summarize the characteristics of the study population: continuous variables were presented as means and standard deviations, and categorical variables as frequencies and percentages.

To examine factors associated with VH, we fitted a multivariable linear regression model with the continuous VH score as the dependent variable. Sociodemographic variables (age, gender, education, place of residence), political sympathies, religious practices, indicators of digital media use (daily Internet and social media use), and psychosocial variables (SHM, VCB, trust in scientists, HL, and eHL) were included as independent variables.

Categorical predictors were modeled using indicator (dummy) coding; for each categorical variable with k categories, k–1 dummy variables were created, with the omitted category serving as the reference group. Coefficients for non-reference categories therefore represent adjusted mean differences in VH relative to the reference category, holding other covariates constant. Reference categories were defined as follows: male gender, rural residence, education level lower than secondary, Law and Justice (ruling party) for political sympathies, practicing religious services more than once per month, 2–3 h of daily Internet use, 30–60 min of daily social media use, and sufficient HL. Model parameters were reported as unstandardized regression coefficients (B) with 95% confidence intervals and *p*-values.

The explanatory power of the model was evaluated using the coefficient of determination (R^2^), adjusted R^2^, and the F-test for overall model significance. Assumptions of linear regression (linearity, homoscedasticity, and normality of residuals) were assessed using residual plots. Bivariate correlations between key variables (Table S1.2), and variance inflation factors were inspected to detect potential multicollinearity among predictors (Table S1.3). All tests were two-tailed, and a *p*-value <0.05 was considered statistically significant.

## 3. Results

### 3.1. Characteristics of the Study Group

The study included 2200 adults (51.1% women, n = 1124) with a mean age of 46.4 years (SD = 15.4). Over one third had lower than secondary education (37.2%, n = 819), while 18.7% (n = 412) held a master’s degree (Table 1). A large share of the sample lived in rural areas (40.5%, n = 892). More than half reported at least four hours of daily Internet use (53.9%, n = 1184), and one third used social media for over an hour per day (33.3%, n = 733). Religious practices were widespread, with 26.7% (n = 587) attending services more than three times per month, while 17.3% (n = 381) identified as non-believers. Politically, the sample broadly reflected national voting patterns, with the ruling Law and Justice party supported by 25.5% (n = 562) of respondents and the main opposition, the Civic Coalition, supported by 22.5% (n = 496).

Health literacy was sufficient in 39.4% (n = 867) of respondents but inadequate in 37.0% (n = 815). Mean scores on key continuous measures were as follows: susceptibility to misinformation 36.91 (SD = 8.71), e-health literacy 29.18 (SD = 5.19), trust in science 40.63 (SD = 7.27), and vaccine conspiracy beliefs 27.12 (SD = 10.44).

### 3.2. Multivariable Linear Regression of Vaccine Hesitancy

The regression model explained 57.8% of the variance in VH (R^2^ = 0.578; adjusted R^2^ = 0.572). The overall model was significant (F(33, 2166) = 89.95), indicating that the set of predictors jointly associated with VH.

The regression analysis revealed several significant predictors of VH (Table 2). Susceptibility to misinformation emerged as a robust positive predictor (B = 0.11, 95% CI: 0.08–0.15), indicating that individuals more vulnerable to misinformation exhibited greater reluctance toward vaccination. Similarly, VCB demonstrated a strong positive association with VH (B = 0.44, 95% CI: 0.41–0.46), highlighting the pivotal influence of conspiratorial narratives in shaping attitudes toward immunization.

In contrast, trust in scientists was inversely related to VH (B = −0.20, 95% CI: −0.23–−0.17), suggesting that greater confidence in scientific expertise corresponded to reduced reluctance. eHL also displayed a negative association (B = −0.07, 95% CI: −0.11–−0.02), implying that individuals with higher levels of digital health competence were less hesitant.

Age exerted a modest yet consistent negative effect (B = −0.02, 95% CI: −0.03–0.00), indicating that older individuals were less prone to hesitancy. Place of residence played a role as well, with inhabitants of small urban areas (<20,000 inhabitants; B = −0.81, 95% CI: −1.51–−0.12) and mid-sized urban centers (200,000–500,000 inhabitants; B = −1.03, 95% CI: −1.82–−0.24) reporting lower levels of VH relative to rural residents. Educational attainment emerged as another determinant: individuals with secondary education (B = −0.58, 95% CI: −1.16–−0.01) and those with a master’s degree (B = −0.77, 95% CI: −1.42–−0.13) were less hesitant compared to participants with education below the secondary level.

Political sympathies further differentiated vaccination attitudes. Respondents classified as “Not eligible or other” exhibited substantially higher VH compared to supporters of the ruling Law and Justice party (B = 1.12, 95% CI: 0.09–2.15).

By contrast, HL, religious practices, gender, Internet use, and social media activity were not significantly associated with VH in the multivariate model.

## 4. Discussion

### 4.1. Overview of Key Findings

This study investigated determinants of adult VH with a focus on psychological and cognitive variables, including SHM, VCB, trust in science, HL and eHL. The multivariable regression model revealed that susceptibility to misinformation and VCB were the positive predictors of VH, while trust in scientists and eHL emerged as protective factors. Several sociodemographic variables (age, education, place of residence) showed smaller but notable associations.

We note that vaccine conspiracy beliefs are conceptually adjacent to vaccine hesitancy in the broader literature and are explicitly included as one component only in extended frameworks such as the 7C model [7], whereas they are not part of the core 3C/5C formulations [5]. Accordingly, the high explained variance should be interpreted cautiously, because part of it is likely driven by shared evaluative content across vaccine-related attitudinal constructs rather than by a purely “unexpected” explanatory gain. Importantly, our outcome (VHS) did not include conspiracy-themed content (e.g., collusion or intentional deception), whereas VCB captured endorsement of such narratives; thus, the constructs are related but not isomorphic. We therefore emphasize the incremental contributions of conceptually distinct mechanisms (e.g., HMS, trust in scientists, and eHL) rather than treating R^2^ as a standalone marker of model excellence.

### 4.2. Susceptibility to Health Misinformation

The robust effect of susceptibility to misinformation underscores the importance of information environments in shaping vaccination intentions. Individuals who scored higher on susceptibility measures were significantly more hesitant to vaccinate, consistent with international findings that misinformation exposure reduces vaccine confidence [34,35]. This vulnerability is not merely a matter of knowledge deficits but reflects cognitive and motivational factors such as low analytical thinking, reliance on intuitive reasoning, and motivated skepticism toward official narratives [50,65]. These mechanisms suggest that interventions cannot rely solely on information provision but must also address the psychological appeal of misinformation, which often provides simple and emotionally resonant explanations.

### 4.3. Vaccine Conspiracy Beliefs

Closely connected is the strong link between conspiracy beliefs and VH. Conspiratorial thinking provides explanatory frameworks that delegitimize scientific evidence and portray vaccines as instruments of control or harm [66,67]. As shown elsewhere, conspiracy beliefs often serve as mediators between susceptibility to misinformation and vaccine refusal [40,68]. This relationship is part of a broader pattern of health denialism, characterized by the systematic rejection of expert consensus and the substitution of pseudo-expert claims [36,69]. Denialist narratives gain traction in online environments where misinformation circulates unchecked, particularly through social media echo chambers [70,71].

### 4.4. Trust in Scientists

Trust in scientists demonstrated a strong protective association, supporting prior evidence that confidence in scientific institutions predicts compliance with health guidance and vaccine uptake [72,73]. However, even in individuals with moderate trust, susceptibility to misinformation can undermine protective effects, suggesting that trust alone is insufficient when misinformation is pervasive and emotionally charged [34,74,75]. This highlights the need for sustained science communication efforts that emphasize transparency, acknowledge uncertainty, and engage with citizens in participatory ways. Trust must be cultivated through dialogue and responsiveness to public concerns rather than one-way transmission of information.

### 4.5. e-Health Literacy

eHL was inversely associated with VH, though its effect was weaker than expected. This result partially contrasts with research highlighting the protective role of digital literacy [76,77,78]. One explanation is that the eHEALS instrument measures self-perceived competencies, which may not fully capture critical evaluation of online content [79,80]. Moreover, motivated reasoning may reduce the capacity of literate individuals to resist misinformation when messages align with their pre-existing beliefs [81]. Nevertheless, the protective role of digital skills, even if modest, indicates that educational interventions promoting critical appraisal of online health information remain worthwhile.

### 4.6. Health Literacy

Interestingly, HL categories were not significant predictors. Some authors have suggested that inadequate HL contributes to vaccine skepticism [82], yet our findings did not confirm this. A plausible explanation is that HL influences general health behaviors more than highly politicized and identity-laden decisions such as vaccination. Hornsey et al. demonstrated that vaccination decisions are not purely health-based. Individuals with conspiratorial worldviews, particularly those harboring suspicion of elites and “the system”, exhibit significantly greater vaccine skepticism [69]. This phenomenon stems from identity protection mechanisms, wherein people with specific ideological orientations (individualism, distrust of authority) fear the loss of autonomy, triggering reactance responses. Consequently, vaccination emerges as a politically and ideologically charged decision rather than a purely health-related one. Notably, education, accounted for nonsignificant variance in vaccination attitudes. It is also possible that traditional literacy measures insufficiently capture the evaluative and trust-related dimensions most relevant to vaccine decision-making. The lack of association between HL and VH may be also related to the way the HLS-EU-Q6 measures HL. It is a short self-report scale and mainly reflects how easy people feel it is to find and understand health information, which is closer to self-efficacy than to objective skills. Therefore, it is still possible that deficits in critical appraisal, e.g., evaluating sources or recognizing misleading claims, influence hesitancy even when self-rated general health literacy is high.

### 4.7. Sociodemographic Variables and Digital Exposure

Among sociodemographic variables, older age was significantly associated with lower hesitancy, in line with studies indicating that younger adults show more skepticism [83]. However, the magnitude of this association was small; thus, while statistically significant, it is likely of limited practical significance. Higher education, particularly a master’s degree, was linked to reduced hesitancy, consistent with previous work [84]. Living in larger urban centers was also associated with lower hesitancy, possibly reflecting greater exposure to pro-vaccine norms, denser health infrastructures, and stronger access to scientific information [85,86]. Gender was not independently associated with hesitancy in the multivariable model.

Regarding digital exposure, the time-based measures of daily Internet and social media use were not significant predictors of hesitancy, despite prior evidence linking online exposure with misinformation susceptibility [87,88,89]. The absence of an independent association may indicate that duration of use is an imprecise proxy that aggregates very heterogeneous online activity, including both reliable information and misleading narratives. Opposing influences may cancel out once SHM, VCB, and trust in scientists are accounted for. In this view, the content and credibility of exposure, rather than quantity, is more relevant for shaping vaccine attitudes. Future studies should use more specific measures of online exposure, such as the main sources used for health information, the types of platforms and accounts followed, and the frequency of encountering and engaging with vaccination content.

### 4.8. Political Sympathies

Similarly, political sympathies and religious practices did not show strong associations in the fully adjusted model. Our earlier study showed a significant relationship between political sympathies and uptake of COVID-19 vaccination [42]. In the present study, we assessed general attitudes toward vaccination and, in the multivariable model, included both susceptibility to misinformation and trust in scientists. These factors may attenuate or mediate the association between political views and VH such that political orientation may operate mainly through more proximal determinants captured in the model (e.g., SHM, VCB, and institutional trust), rather than exerting a strong direct effect on hesitancy once these constructs are included. This null pattern is therefore informative. It suggests limited incremental explanatory value of political sympathies after accounting for these psychological and informational factors, although the precision of some category estimates was limited (wide confidence intervals). It should be emphasized, however, that in highly polarized societies, both political polarization [90,91] and religious identity [92,93] have been shown to be significant predictors of vaccination attitudes and the relevance of political and religious factors may be context-specific and moderated by the intensity of polarization and the local information environment.

We observed comparatively higher VH in the combined “Not eligible or other” category relative to Law and Justice, which served as the reference group. “Not eligible” referred mainly to respondents who reported being unable to vote in the most recent elections (typically those who had not yet reached voting age at that time), whereas “other” captured supporters of small parties and coalitions not listed separately. The categories were combined due to small cell sizes to avoid unstable estimates, which limits substantive interpretation. Importantly, because age was included as a covariate in the multivariable model, this residual association should not be interpreted as merely reflecting youth. Instead, it may capture heterogeneous, age-independent correlates of this residual category (e.g., weakly anchored political identification, political disengagement, or lower perceived institutional efficacy), although these interpretations remain speculative. Given the wide confidence interval and the heterogeneity of the combined category, we interpret this estimate cautiously and suggest that future surveys should distinguish these subgroups more precisely. Importantly, Confederation, modeled separately as the extreme right-wing party, did not differ from the reference group

### 4.9. Religious Practices

Religious practices showed no independent association with VH after adjustment for other covariates. Attendance is also a distal marker, and its relationship with VH may be largely accounted for by more proximal factors included in the model, especially trust in scientists, SHM and VCB. It is also possible that attendance frequency is too broad to capture dimensions of religiosity more directly linked to vaccination attitudes, such as religious identity, community norms, or vaccine-related messages within religious networks.

### 4.10. Implications of the Study

Our findings carry several important implications. First, susceptibility to misinformation should be recognized as a distinct psychological construct to be routinely assessed in public health monitoring. This can help identify groups most at risk of hesitancy and guide targeted interventions. Second, public health communication should move beyond reactive fact-checking and incorporate prebunking strategies that “inoculate” individuals against persuasive falsehoods before they are encountered [94,95]. Interactive educational campaigns and gamified interventions have shown promise in building such resilience. Third, efforts to build trust in science should emphasize openness and humility, recognizing that distrust is often rooted in broader social and political grievances. Initiatives that involve community leaders, health professionals, and local institutions may be particularly effective. Fourth, educational systems should integrate critical media literacy and digital health literacy into curricula, equipping citizens with long-term skills to evaluate online health information. Finally, social media platforms and regulators should continue developing policies that reduce the algorithmic amplification of harmful misinformation, while fostering visibility of credible, accessible health information. Together, these implications highlight the multifaceted challenge of VH. It is not solely a question of access to information, but of cognitive vulnerability, social identity, and trust in institutions. Addressing these issues requires coordinated action across education, communication, and regulation.

### 4.11. Limitations

This study has several limitations. Due to the cross-sectional design, causal direction cannot be established and bidirectional relationships are possible. Vaccine-hesitant individuals may be more likely to endorse misinformation and conspiratorial narratives to justify prior attitudes, while acceptance of such content may also increase hesitancy over time. Therefore, findings should be interpreted as associations rather than effects. Reliance on self-reported data raises risks of bias, including social desirability and self-assessment inaccuracies. Although the sample was nationally representative, it was limited to a single country, which restricts generalizability across cultural contexts. eHL was assessed through self-perceived competence rather than objective skills, which may have led to an underestimation of its role. The study also did not account for the content or sources of online exposure, which may shape misinformation susceptibility in nuanced ways. In addition, political and cultural polarization may moderate the observed effects, and these contextual dynamics were not fully captured.

Because data were collected via web-based survey, adults who do not use the Internet were not represented. This digital divide coverage bias may affect estimates of VH and trust, particularly among older and socioeconomically disadvantaged groups, and it may partly account for the observed lower VH with age as the online sample captures a more digitally engaged subgroup of seniors. At the same time, Internet use increases exposure to vaccine misinformation and polarized content, which may push hesitancy upward among online respondents and partially counterbalance this bias. Therefore, results should be generalized primarily to the Internet-using adult population.

Finally, the Susceptibility to Health Misinformation (SHM) measure was intentionally anchored in specific misinformation narratives circulating in Poland at the time of data collection (2024) to maximize ecological validity. As the information environment evolves and particular news stories fade from public attention, some items may lose salience, which can reduce temporal stability and implies that periodic item updating and revalidation may be needed.

## 5. Conclusions

This study highlights SHM as a central predictor of VH, operating both directly and through endorsement of conspiracy beliefs and broader health denialist narratives. The findings confirm that individuals more vulnerable to misleading information are significantly less likely to accept vaccination, even when they report adequate levels of health literacy or general trust in science. Importantly, while e-health literacy and trust in scientists were protective factors, their effects were weaker than those of misinformation susceptibility, suggesting that information-processing vulnerabilities can override knowledge and confidence in institutions when misinformation is pervasive and emotionally persuasive.

The results underscore the need for multifaceted strategies to address VH. Public health communication should not only correct false claims but also strengthen citizens’ resilience to misleading narratives through prebunking and inoculation approaches. Enhancing digital and critical media literacy remains essential, yet interventions must go beyond individual skills and account for the emotional, motivational, and social identity dynamics that make misinformation persuasive. Building and maintaining trust in scientific and health institutions is equally crucial, requiring transparency, responsiveness, and active engagement with communities.

Practically, policymakers and health authorities should prioritize monitoring susceptibility to misinformation as part of vaccine confidence surveillance, develop evidence-based educational programs that foster analytical thinking, and collaborate with social media platforms to limit amplification of harmful content. Efforts should be tailored to demographic groups identified as more hesitant, such as younger individuals or those in rural settings, while also addressing the broader sociopolitical contexts that fuel denialism.

In sum, combating VH demands a holistic approach that integrates psychological insights with communication, education, and policy measures. Only by addressing both the cognitive vulnerabilities and the socio-political conditions enabling misinformation can long-term vaccine confidence be secured.

## Figures and Tables

**Table 1:** Characteristics of the study group.

Variable	Categories	%	n
Gender	male	48.9	1076
	female	51.1	1124
Education	lower than secondary	37.2	819
	secondary	23.4	514
	postsecondary non-university	11.0	243
	university Bachelors’	9.6	212
	university Masters	18.7	412
Place of residence	rural	40.5	892
	urban < 10.000	13.4	294
	urban 10,000–100,000	18.6	409
	urban 100,000–200,000	8.7	191
	urban 200,000–500,000	9.9	218
	urban > 500,000	8.9	196
Internet daily use	<1 h	7.5	165
	1–3 h	38.7	851
	4–5 h	29.4	646
	>5 h	24.5	538
Social media daily use	<15 min	20.2	444
	15–30 min	22.2	488
	30–60 min	24.3	535
	>60 min	33.3	733
Political	Low and Justice	25.5	562
	Civic Coalition	22.5	496
	Poland2050	10.3	226
	The New Left	6.2	137
	Confederation Freedom and Independence	5.4	119
	non-voters	24.3	535
	other or not eligible	5.7	125
Participation in religious practices	non-believers	17.3	381
	not practicing believers	34.6	761
	1–3 monthly	21.4	471
	>3 monthly	26.7	587
Health literacy	problematic	8.7	191
	inadequate	37.0	815
	sufficient	39.4	867
	undetermined	14.9	327

**Table 2:** Multivariable linear regression model of adult vaccination hesitancy.

Variable	Categories of Variables	B(SE)	Lower 95% CI	Upper 95%CI	*p*
Susceptibility to misinformation		0.11 (0.02)	0.08	0.15	<0.001
Vaccine conspiracy beliefs score		0.44 (0.01)	0.41	0.46	<0.001
Trust in scientists		−0.20 (0.02)	−0.23	−0.17	<0.001
E-health literacy		−0.07 (0.02)	−0.11	−0.02	0.006
Health literacy	Sufficient #				
	Inadequate	−0.41 (0.44)	−1.27	0.45	0.352
	Problematic	0.17 (0.27)	−0.37	0.70	0.538
	Undetermined	−0.36 (0.35)	−1.05	0.34	0.314
Daily Internet use	2–3 h #				
	≤1 h	−0.03 (0.45)	−0.91	0.85	0.943
	4–5 h	0.37 (0.28)	−0.18	0.93	0.185
	>5 h	0.22 (0.31)	−0.38	0.83	0.475
Daily use of social media	30–60 min #				
	<15 min or no use	0.25 (0.35)	−0.43	0.93	0.467
	15–30 min	−0.21 (0.33)	−0.85	0.44	0.531
	>60 min	−0.49 (0.31)	−1.09	0.11	0.110
Age		−0.02 (0.01)	−0.03	−0.01	0.025
Gender	Male #				
	Female	−0.34 (0.23)	−0.79	0.11	0.137
Place of residence	Rural #				
	Urban < 20,000	−0.81 (0.35)	−1.51	−0.12	0.021
	Urban 20,000–100,000	−0.50 (0.32)	−1.12	0.11	0.110
	Urban 100,000–200,000	−0.15 (0.42)	−0.97	0.68	0.727
	Urban 200,000–500,000	−1.03 (0.40)	−1.82	−0.24	0.011
	Urban > 500,000	−0.47 (0.42)	−1.29	0.36	0.266
Education level	lower than secondary #				
	secondary	−0.58 (0.29)	−1.16	−0.01	0.047
	post-secondary non-university	−0.56 (0.38)	−1.31	0.19	0.143
	university Bachelors	−0.53 (0.41)	−1.33	0.27	0.191
	university Masters	−0.77 (0.33)	−1.42	−0.13	0.018
Political sympathies	Law and Justice (ruling party) #				
	Civic Coalition	−0.28 (0.34)	−0.94	0.38	0.398
	Poland 2050	0.32 (0.42)	−0.50	1.13	0.445
	The New Left	−0.15 (0.5)	−1.13	0.83	0.760
	Confederation	0.39 (0.53)	−0.66	1.44	0.465
	Non-voters	0.45 (0.32)	−0.17	1.07	0.157
	Not eligible or other	1.12 (0.52)	0.09	2.15	0.033
Religious practices	>1 monthly #				
	Non-believers	0.39 (0.36)	−0.31	1.09	0.271
	Not practicing believers	−0.12 (0.29)	−0.70	0.45	0.670
	≤1 monthly	−0.36 (0.32)	−0.31	1.09	0.263

#—reference category of the categorical variable

## Data Availability

The dataset generated and analyzed during the current study is available in the ZENODO repository—doi:10.5281/zenodo.17782003 (available at https://zenodo.org/uploads/17782003, accessed on 12 February 2026).

## Supplementary Materials

The following supporting information can be downloaded at: https://www.mdpi.com/article/10.3390/healthcare14040497/s1: Supplementary File S1 containing Table S1.1: The list of statements included in the Susceptibility to Misinformation Instrument and their sources, Table S1.2: Correlation matrix, Table S1.3: Multicollinearity testing results, and Supplementary File S2 comprising factorial validation of the HMSI (polychoric EFA, forced one-factor EFA, and benchmark CFA models, Table S2.1: EFA diagnostics and factorability (polychoric correlations), Table S2.2: Three-factor EFA variance accounted for by each factor, Table S2.3: Three-factor EFA standardized pattern matrix and communalities (MINRES, oblimin), Table S2.4: Three-factor EFA global fit indices and reliability, Table S2.5: Three-factor EFA factor correlations, Table S2.6: Forced one-factor EFA loadings and communalities, Table S2.7: Forced one-factor EFA global indices and reliability, Table S2.8: CFA model fit indices (one-factor vs three-factor), Table S2.9: CFA standardized loadings (one-factor and three-factor), Table S2.10: CFA factor correlations in the benchmark three-factor model).

## Abbreviations

The following abbreviations are used in this manuscript:

aVHS	Adult Vaccine Hesitancy Scale
eHEALS	e-Health Literacy Scale
HLS-EU-Q6	6-item Health Literacy Survey European Questionnaire
HMSI	Health Misinformation Susceptibility Instrument
VCBS	Vaccine Conspiracy Beliefs Scale
VH	Vaccine Hesitancy
