# Supplementary material for: Exploring the Relationship Between Susceptibility to Health Misinformation and Vaccine Hesitancy in Poland
Source: Healthcare (Basel). 2026 Feb 14;14(4):497. doi: 10.3390/healthcare14040497 (PMC12941334; doi:10.3390/healthcare14040497)
Supplement: Supplementary file 1 [file healthcare-14-00497-s001.zip › Supplementary File 1.pdf]

Table S1.1. The list of statements included in the Susceptibility to Misinformation Instrument and their sources.

Polish version of the Item	English version of the item	Primary fact-checking service	Publication date	Link to fact-checking website	Other fact-checking services addressing the topic of the statement
(FALSE1) Szczepionki przeciw HPV zawierają wirusy	HPV vaccines contain viruses	Fakenews.pl	June 23, 2023	https://fakenews.pl/zdrowie/szczepionki-przeciw-hpv-nie-zawieraja-wirusow-komorek-owadow-ani-rakotworczych-bialek/	Szczepienia.info, May 31, 2023, https://szczepienia.pzh.gov.pl/dla-lekarzy/szczepienia-hpv/sklad-i-jakosc-szczepionek-przeciw-hpv/
(FALSE2) Islandia zakazała szczepień przeciw COVID-19 ze względu na nadmiarową liczbę zgonów	Iceland has banned COVID-19 vaccinations due to the excess number of deaths	Konkret24	December 10, 2023	https://konkret24.tvn24.pl/zdrowie/covid-19-fake-newsy-o-zabojczych-szczepionkach-wyjasniamy-st7520666	Fakenews.pl, December 11, 2023, https://fakenews.pl/zdrowie/islandia-nie-zakazala-szczepien-przeciw-covid-19/
(FALSE3) Ekspozycja na promieniowanie rentgenowskie w trackie badania mammograficznego może pobudzać wzrost guza piersi i pojawienie się przerzutów	Exposure to X-ray radiation during a mammographic examination may stimulate the growth of a breast tumor and the development of metastases	Demagog.pl	November 10, 2023	https://demagog.org.pl/fake_news/czy-mammografia-jest-niebezpieczna-dla-kobiet-wyjasniamy/	Serwis Zdrowie, May 19, 2023 https://zdrowie.pap.pl/byc-zdrowym/mammografia-nie-warto-obawiac-sie-promieniowania
(FALSE4) Chityna zawarta w szkieletach zewnętrznych owadów jest szkodliwa dla zdrowia	Chitin contained in the exoskeletons of insects is harmful to human health.	Demagog.pl	February 9, 2023	https://demagog.org.pl/fake_news/jadalne-owady-sa-szkodliwe-dla-zdrowia-ludzi-wyjasniamy/	Fakenews.pl, March 2, 2023, https://fakenews.pl/zdrowie/chityna-jest-substancja-bezpieczna-dla-czlowieka/
(FALSE5) Ze względu na wojnę na Ukrainie zezwolono na przeszczepy organów bez gody dawców	Due to the war in Ukraine, organ transplants without the donors' consent have been permitted.	Demagog.pl	June 21, 2024	https://demagog.org.pl/fake_news/zelenski-zezwolil-na-przeszczepy-organow-bez-zgody-dawcy-falsz/	
(FALSE6) Wyciąg z muchomora pobudza regenerację neuronów	An extract from the fly agaric mushroom stimulates the regeneration of neurons.	Demagog.pl	October 10, 2024	https://demagog.org.pl/fake_news/muchomor-nie-odbuduje-neuronow-uwaga-na-falszywe-zalecenia/	
(FALSE7) Probiotyki i witaminy hamują rozwój posocznicy	Probiotics and vitamins inhibit the development of sepsis.	Demagog.pl	October 9, 2024	https://demagog.org.pl/fake_news/probiotyki-i-witaminy-lecza-raka-oraz-sepse-nauka-mowi-co-innego/	
(FALSE8) Duże dawki witaminy C są skuteczne w leczeniu nowotworów u człowieka, m.in. raka prostaty i trzustki	High doses of vitamin C are effective in treating cancers in humans, including prostate and pancreatic cancer.	Newsweek.pl	May 19, 2023	https://www.newsweek.pl/polska/spoleczenstwo/dezinformacja-jerzy-zieba-i-lewoskretna-witamina-c/k4c3y0d	PAP, Serwis Zdrowie, October 21, 2021, https://zdrowie.pap.pl/dieta/suplementy-na-raka-nie-dajcie-sie-nabrac
(FALSE9) Technologia 5G przyczynia się do zmniejszenia płodności u ludzi	5G technology contributes to reduced fertility in humans.	Demagog.pl	September 25, 2024	https://demagog.org.pl/fake_news/5g-grozne-dla-ludzkosci-wyjasniamy-falszywe-tezy-pseudonaukowca/	Fakenews.pl, October 4, 2023, https://fakenews.pl/technologia/testy-systemow-alarmowych-4-pazdziernika-w-usa-nie-beda-sluzyc-depopulacji/

(FALSE10) Słońce pobudza wytwarzanie naturalnych szczepionek	The sun stimulates the production of natural vaccines.	Demagog.pl	September 12, 2024	https://demagog.org.pl/fake_news/slonce-produkuje-naturalne-szczepionki-to-dezinformacja/	
(FALSE11) Firma Moderna przyznała, że produkowane przez nią szczepionki mogą powodować raka	The company Moderna has admitted that the vaccines it produces may cause cancer.	Konkret24.tvn24.pl	December 2, 2023	https://konkret24.tvn24.pl/zdrowie/koronawirus-turbo-rak-po-szczepieniach-na-covid-19-skad-sie-wzial-ten-fake-news-st7460496	PAP, September 17, 2024, Serwis Zdrowie, https://zdrowie.pap.pl/byc-zdrowym/szczepienia-na-covid-19-nie-prowadza-do-turboraka
(FALSE12) Małpia ospa jest efektem ubocznym szczepień przeciwko COVID-19	Monkeypox is a side effect of COVID-19 vaccination.	Termedia	May 25, 2022	https://www.termedia.pl/poz/To-nieprawda-ze-malpia-ospa-byla-w-szczepionce-przeciw-COVID-19-prof-Pyrc-dementuje-fake-newsy,47180.html	Demagog.pl, September 6, 2024, https://demagog.org.pl/fake_news/co-laczy-malpia-ospe-polpasiec-i-szczepienia-sprawdzamy-fakty/

Table S1.2. Pearson and Spearman correlations between key constructs (N=2200)

Variable 1	Variable 2	Pearson r	Spearman ρ
Susceptibility to health misinformation score	Vaccine conspiracy beliefs score	0.57***	0.55***
Susceptibility to health misinformation score	Trust in scientists score	-0.19***	-0.19***
Susceptibility to health misinformation score	E-health literacy score	0	-0.04*
Vaccine conspiracy beliefs score	Trust in scientists score	-0.34***	-0.33***
Vaccine conspiracy beliefs score	E-health literacy score	0.01	0.01
Trust in scientists score	E-health literacy score	0.25***	0.27***

Significance codes: * p<0,05; ** p<0,01; *** p<0,001 (two-tailed).

Table S1.3. Multicollinearity testing

Variable	Category	Tolerance	VIF
Susceptibility to misinformation		0.643	1.555
Vaccine conspiracy beliefs score		0.586	1.706
Trust in scientists		0.796	1.256
E-health literacy		0.781	1.280
Health literacy	Inadequate	0.788	1.268
	Problematic	0.701	1.427
	Undetermined	0.759	1.318
Political sympathies	Civic Coalition	0.609	1.641
	Poland 2050	0.755	1.324
	The New Left	0.827	1.210
	Confederation	0.827	1.209
	Non-voters	0.652	1.535
	Not eligible or other	0.817	1.224
Religious practices	Non-believers	0.663	1.508
	Not practicing believers	0.626	1.598
	≤1 monthly	0.683	1.465
Age		0.777	1.286
Gender	Female	0.917	1.090
Place of residence	Urban <20,000	0.836	1.196
	Urban 20,000–100,000	0.802	1.247
	Urban 100,000–200,000	0.858	1.165
	Urban 200,000–500,000	0.827	1.210
	Urban >500,000	0.842	1.188
Education level	Secondary	0.783	1.277
	Post-secondary non-university	0.844	1.185
	University Bachelors	0.836	1.196
	University Masters	0.735	1.360
Daily use of social media	<15 minutes or no use	0.625	1.601
	15–30 minutes	0.648	1.543
	>60 minutes	0.579	1.726
Daily Internet use	≤1 hour	0.861	1.162
	4–5 hours	0.733	1.364
	>5 hours	0.684	1.462
