# Supplementary material for: Exploring the Relationship Between Susceptibility to Health Misinformation and Vaccine Hesitancy in Poland
Source: Healthcare (Basel). 2026 Feb 14;14(4):497. doi: 10.3390/healthcare14040497 (PMC12941334; doi:10.3390/healthcare14040497)
Supplement: Supplementary file 1 [file healthcare-14-00497-s001.zip › Supplementary File 2.pdf]

Supplementary Material File 2. Structural validity of the Health Misinformation Susceptibility Instrument

The analyses below were added in response to a reviewer request to document the factorial structure of the ad hoc Health Misinformation Susceptibility Instrument (HMSI) and to clarify item scoring.

Methods

The Health Misinformation Susceptibility Instrument (HMSI) comprises 12 health-related items (item1–item12, please see Supplementary Material File 1, Table S.1) evaluated on a 6-point forced-choice agreement scale from 1 (decidedly untrue) to 6 (decidedly true), with no neutral midpoint. Because all statements are factually incorrect, higher endorsement reflects greater susceptibility to misinformation. A single-score index was operationalized as the sum of the 12 item responses (range 12-72).

The HMSI indicators were treated as ordered-categorical. In this setting, linear associations based on Pearson correlations can be attenuated or distorted, particularly when response distributions are skewed or when the ordinal thresholds are uneven. Therefore, exploratory analyses were based on polychoric correlations, which approximate the association between latent continuous response propensities assumed to generate the observed ordinal categories through thresholding. This approach is recommended for recovering latent structure from Likert-type items when the primary goal is dimensionality assessment rather than treating the indicators as truly continuous.

To reduce the risk of overfitting and to separate exploration from confirmation, the available dataset was partitioned into two non-overlapping subsets: an exploratory factor analysis (EFA) subset (n=1121) and a confirmatory factor analysis (CFA) subset (n=1079).

EFA was conducted in the dedicated subset (n=1121) using the polychoric correlation matrix as input. Prior to factor extraction, factorability and numerical suitability of the matrix were evaluated using complementary diagnostics, including item-level response distributions (missingness and empty categories), correlation-matrix properties relevant for stable extraction (including positive definiteness and eigenvalue structure), and standard sampling-adequacy and sphericity tests (Kaiser–Meyer–Olkin measure, including item-level MSA, and Bartlett's test of sphericity). Factor extraction used minimum residual estimation (MINRES), a correlation-based method that is computationally stable and does not depend on strict multivariate normality assumptions for the observed ordinal indicators.

Because substantive components of misinformation endorsement (if present) were expected to share common variance rather than be strictly independent, an oblique rotation was applied. Specifically, oblimin rotation was used to allow inter-factor correlations and to avoid imposing orthogonality that would be theoretically implausible in this context.

The number of factors was guided by parallel analysis implemented for polychoric correlations, which compares observed eigenvalues to those expected under random data and is generally preferred over heuristic rules (e.g., Kaiser's eigenvalue>1). In addition to the empirically suggested multi-factor solution, a forced one-factor EFA was also estimated to evaluate the extent to which a dominant general factor could account for common variance when multidimensionality was constrained and to provide one-factor loadings and reliability indices aligned with the intended use of a single composite score.

CFA was conducted in the independent subset (n=1079) to cross-validate dimensionality. A one-factor model (target) specified all items loading on a single latent susceptibility factor. A three-factor model (benchmark) followed the clustering observed in EFA and allowed the latent factors to correlate. Model fit was evaluated using χ^2, CFI, TLI, RMSEA with confidence intervals, and SRMR. Preference for a single-score solution was supported when the one-factor model demonstrated acceptable global fit and when the three-factor benchmark yielded high inter-factor correlations, indicating limited discriminant separation among the specific factors.

All analyses were performed in IBM SPSS Statistics v29 using the embedded R environment (BEGIN PROGRAM R). Item responses were transferred from SPSS to R via the SPSS–R integration interface (`spssdata.GetDataFromSPSS`). Polychoric correlations, exploratory factor analysis (EFA), factorability diagnostics (KMO, Bartlett's test), parallel analysis, and ordinal reliability indices were computed in R using the `psych` package (including MINRES extraction and oblimin rotation for multifactor solutions). Confirmatory factor analysis (CFA) was estimated in `lavaan` using an ordinal WLSMV estimator (parameterization="theta" with items declared as ordered), comparing a unidimensional target model to a correlated three-factor benchmark model aligned with the exploratory pattern; model evaluation relied on χ^2 and standard global fit indices (CFI, TLI, RMSEA with 90% CI, SRMR).

Results

EFA diagnostics supported factorability of the HMSI polychoric correlation matrix (Table S2.1). Sampling adequacy was high (KMO=0.902), and Bartlett's test of sphericity was statistically significant ($\chi^2(66)=3158.056$, $p<0.001$). Parallel analysis suggested a three-factor solution.

Table S.2.1. EFA diagnostics and factorability (polychoric correlations)

**Metric**	**Result**
EFA subset size (n)	1121
Missing data per item (%)	0 (all items)
Empty response categories (1-6)	none (all categories used)
Determinant of polychoric R	0.0589
KMO overall (MSA)	0.902
MSA range across items	0.811–0.937

Bartlett's test of sphericity	$\chi^2(66)=3158.056$, $p<0.001$
Proportion of |r_poly| ≥ 0.30	0.455
Parallel analysis suggested	3 factors

The three-factor EFA solution accounted for a total of 38.1% of the variance in the polychoric correlation matrix (cumulative proportion), with the first factor explaining 21.0%, the second 9.1%, and the third 8.0% (Table S2.2).

Table S2.2. Three-factor EFA variance accounted for by each factor.

Index	EFA factor 1	EFA factor 2	EFA factor 3
Sum of squared loadings	2.516	1.097	0.961
Proportion Variance	0.21	0.091	0.08
Cumulative Variance	0.21	0.301	0.381
Proportion Explained	0.55	0.24	0.21
Cumulative Proportion	0.55	0.79	1.0

In the three-factor EFA, item loadings formed three correlated clusters (Table S2.3). Although the three-factor solution provided excellent EFA fit indices (Table S2.4), factor correlations were non-trivial (Factor 1-Factor 3=0.687; Factor 1-Factor 2=0.489; Factor 2-Factor 3=0.297; Table S2.5), indicating shared variance across the putative dimensions.

Table S2.3. Three-factor EFA standardized pattern matrix and communalities (MINRES, oblimin).

Item	EFA factor 1	EFA factor 2	EFA factor 3	Communality (h2)
item1	0.14	0.26	0.04	0.15
item2	0.33	0.43	-0.02	0.48
item3	0.55	0.04	0.05	0.37
item4	0.33	0.09	-0.02	0.15
item5	0.6	0.05	-0.03	0.39
item6	0.59	-0.1	0.12	0.36
item7	-0.04	0.0	0.69	0.45
item8	0.3	0.05	0.4	0.41
item9	0.65	0.04	-0.03	0.45
item10	0.05	0.1	0.44	0.27
item11	-0.02	0.79	0.03	0.61
item12	0.7	0.0	-0.01	0.48

Table S2.4. Three-factor EFA global fit indices and reliability.

Index	Value
RMSR	0.0193
RMSEA (90% CI)	0.029 [0.018, 0.039]
TLI	0.980
BIC	-168.608

Omega total (polychoric)	0.851

Table S2.5. Three-factor EFA factor correlations.

Factor pair	Correlation
Factor 1 – Factor 3	0.687
Factor 1 – Factor 2	0.489
Factor 2 – Factor 3	0.297

When a one-factor structure was forced in EFA, standardized loadings remained mostly moderate (approximately 0.36–0.67) and internal consistency estimates based on polychoric correlations were satisfactory (ordinal alpha=0.827; omega total=0.831; Tables S2.6–S2.7). As expected, one-factor fit was weaker than the unconstrained three-factor EFA, but remained compatible with a dominant general factor representation of the HMSI.

Table S2.6. Forced one-factor EFA loadings and communalities.

Item	Loading	Communality
item1	0.371	0.138
item2	0.65	0.422
item3	0.605	0.366
item4	0.385	0.148
item5	0.608	0.37
item6	0.569	0.324
item7	0.355	0.126
item8	0.572	0.327
item9	0.651	0.424
item10	0.399	0.159
item11	0.587	0.345
item12	0.67	0.449

Table S2.7. Forced one-factor EFA global indices and reliability.

**Index**	**Value**
Sum of squared loadings	3.598
Proportion variance	0.300
RMSR	0.049
RMSEA (90% CI)	0.065 [0.058, 0.072]
TLI	0.900
BIC	-71.455
Omega total (polychoric)	0.831
Ordinal alpha (polychoric)	0.827

In the validation subsample (N=1,079), the ordinal one-factor CFA demonstrated good fit ($\chi^2(54)=259.39$, $p<.001$; CFI=0.982; TLI=0.978; RMSEA=0.059, 90% CI 0.052–0.067; SRMR=0.043) (Table S2.8). Standardized loadings were all statistically significant and ranged from 0.315 to 0.716 (Table S2.9), indicating that each item contributed

meaningfully to a common susceptibility factor. For benchmarking, a correlated three-factor CFA yielded superior fit ($\chi^2(51)=117.38$, $p<.001$; CFI=0.994; TLI=0.993; RMSEA=0.035, 90% CI 0.026–0.043; SRMR=0.031), consistent with the EFA-derived clustering of items (Table S.2.8).

Table S2.8. CFA model fit indices (one-factor vs three-factor).

Model	χ^2	df	p	CFI	TLI	RMSEA (90% CI)	SRMR
1-factor	259.394	54	<0.001	0.982	0.978	0.059 (0.052, 0.067)	0.043
3-factor	117.379	51	<0.001	0.994	0.993	0.035 (0.026, 0.043)	0.031

Table S.2.9. CFA standardized loadings (one-factor and three-factor).

Item	CFA 1-factor	CFA 3-factor: Factor 1	CFA 3-factor: Factor 2	CFA 3-factor: Factor 3
item1	0.315			0.336
item2	0.670			0.73
item3	0.596	0.608		
item4	0.470	0.481		
item5	0.572	0.584		
item6	0.525	0.534		
item7	0.423		0.503	
item8	0.570		0.704	
item9	0.605	0.617		
item10	0.444		0.536	
item11	0.695			0.763
item12	0.716	0.734		

However, latent factor correlations were high (r=0.636–0.861) (Table S2.10), implying that the apparent subdimensions shared substantial variance and exhibited limited discriminant separation. In this context, the incremental improvement in global fit for the three-factor specification was interpreted primarily as capturing residual covariation among closely related item clusters rather than reflecting clearly distinct constructs. Given the satisfactory fit of the unidimensional model, the coherent and uniformly significant loading pattern, the strong inter-factor dependencies observed in the benchmark model, and the intended analytic use of a single HMSI susceptibility score in subsequent regression models, a one-factor representation was retained as the most parsimonious and substantively appropriate parameterization of the instrument.

Table S.2.10. CFA factor correlations in the benchmark three-factor model.

**Factor pair**	**Correlation**
Factor 1 – Factor 2	0.767
Factor 1 – Factor 3	0.861
Factor 2 – Factor 3	0.636
